# *Staphylococcus aureus* Bloodstream Infection and Endocarditis - A Prospective Cohort Study

**DOI:** 10.1371/journal.pone.0127385

**Published:** 2015-05-28

**Authors:** Vincent Le Moing, François Alla, Thanh Doco-Lecompte, François Delahaye, Lionel Piroth, Catherine Chirouze, Pierre Tattevin, Jean-Philippe Lavigne, Marie-Line Erpelding, Bruno Hoen, François Vandenesch, Xavier Duval, VIRSTA study group

**Affiliations:** 1 Maladies infectieuses, CHU de Montpellier, UMI 233 Université Montpellier 1, Institut de Recherche pour le Développement, Montpellier, France; 2 Université de Lorraine, Université Paris Descartes, Apemac, EA 4360, INSERM, CIC-EC, CIE6, CHU Nancy, Nancy, France; 3 CHU de Nancy, Nancy, France; 4 Hospices Civils de Lyon, Université Claude Bernard, Lyon, France; 5 CHU de Dijon, UMR 1347-MERS, Université de Bourgogne, Dijon, France; 6 UMR CNRS 6249 Chrono-environnement, Université de Franche-Comté, CHU de Besançon, Besançon, France; 7 Hôpital Pontchaillou, INSERM U835, Faculté de Médecine, Université Rennes 1, IFR140, Rennes, France; 8 CHU Carémeau, INSERM U1047, Université Montpellier 1, Nîmes, France; 9 Université des Antilles et de la Guyane, Faculté de Médecine Hyacinthe Bastaraud, Pointe-à-Pitre, Guadeloupe, Service des maladies infectieuses, CHU, Pointe-à-Pitre, Guadeloupe, France; 10 Centre National de Référence des Staphylocoques, Hospices Civils de Lyon, Centre International de Recherche en Infectiologie, INSERM U1111, Université Lyon 1, CNRS, UMR 5308, Lyon, France; 11 Université Paris Diderot, INSERM, UMR 1137, CIC 1425, AP-HP, Hôpital Bichat Claude Bernard, Paris, France; University of Lausanne, SWITZERLAND

## Abstract

**Objectives:**

To update the epidemiology of *S*. *aureus *bloodstream infection (SAB) in a high-income country and its link with infective endocarditis (IE).

**Methods:**

All consecutive adult patients with incident SAB (n = 2008) were prospectively enrolled between 2009 and 2011 in 8 university hospitals in France.

**Results:**

SAB was nosocomial in 54%, non-nosocomial healthcare related in 18% and community-acquired in 26%. Methicillin resistance was present in 19% of isolates. SAB Incidence of nosocomial SAB was 0.159/1000 patients-days of hospitalization (95% confidence interval [CI] 0.111-0.219). A deep focus of infection was detected in 37%, the two most frequent were IE (11%) and pneumonia (8%). The higher rates of IE were observed in injecting drug users (IE: 38%) and patients with prosthetic (IE: 33%) or native valve disease (IE: 20%) but 40% of IE occurred in patients without heart disease nor injecting drug use. IE was more frequent in case of community-acquired (IE: 21%, adjusted odds-ratio (aOR) = 2.9, CI = 2.0-4.3) or non-nosocomial healthcare-related SAB (IE: 12%, aOR = 2.3, CI = 1.4-3.5). *S*. *aureus* meningitis (IE: 59%), persistent bacteremia at 48 hours (IE: 25%) and C-reactive protein > 190 mg/L (IE: 15%) were also independently associated with IE. Criteria for severe sepsis or septic shock were met in 30% of SAB without IE (overall in hospital mortality rate 24%) and in 51% of IE (overall in hospital mortality rate 35%).

**Conclusion:**

SAB is still a severe disease, mostly related to healthcare in a high-income country. IE is the most frequent complication and occurs frequently in patients without known predisposing conditions.

## Introduction

Staphylococcus aureus is a common cause of bloodstream infection in high- income countries [1,2], with an estimated incidence between 80 and 190 cases / 100.000 inhabitants per year. This would represent an estimated 600.000 cases per year in the USA and 125.000 in France [2]. *S*. *aureus* bloodstream infection (SAB) incidence may be increasing, at least in some regions [3–5], probably due to higher numbers of invasive procedures and/or at-risk situations, including the presence of endovascular devices. In recent series, SAB was associated with high morbidity and a 20% or more case fatality rate [6]. However, large prospective epidemiological studies of SAB have been rare since 2000 [7–12].

SAB represents two distinct clinical entities that differ in terms of management and prognosis [1]: SAB without any detected deep focus of infection for which a treatment duration of 14 days is recommended, and those associated with a deep infectious focus (whether cause or consequence of bacteremia) which represent 40 to 70% of SAB cases and should be treated for 4 to 6 weeks. The most frequent reported deep infectious foci are endovascular foci including infective endocarditis (IE), central nervous system, bone and joint, soft tissue and intra abdominal/pelvic infections.

Among SAB with a deep focus of infection, those associated with IE are of particular interest since they are the most frequent and have the poorest prognosis whether endovascular infection be the cause or the consequence of SAB. Furthermore, *S*. *aureus* has become, during the last decade, the leading cause of IE in Western Europe and North America [13–15], and a microorganism clearly identified in IE patients, as responsible for the highest rates of morbidity and mortality [14–16]. Frequency of IE in SAB patients varies according to studies from 10 to 46%, likely due to varying proportions of subjects with IE risk factors in these populations (e.g. injecting drug use, prosthetic valve or underlying heart disease) [17]. Detection of IE in SAB patients leads to adaptation of antibiotic therapy doses and duration. Early detection of IE may also be beneficial by allowing early heart surgery when necessary [18]. The systematic use of echocardiography in SAB remains however a matter of debate [1,19–21].

We conducted a large prospective exhaustive cohort study in 8 university hospitals in France during a 30-month period, to (i) update the information on the epidemiology of SAB in a high-income country, (ii) evaluate the frequency of different deep foci of infection including IE and (iii) identify factors associated with IE in case of SAB.

## Materials and Methods

### Ethics

The “Comité de Protection des Personnes Sud-Méditerranée IV” approved the study. The VIRSTA study is registered in the European Clinical Trials Database (EUDRACT) under the number: 2008-A00680-55. According to French legislation, and because no intervention was performed on patients, no written informed consent was given by the patients. Patients records and informations were anonymized and de-identified prior to analyses.

### Study population—collected data

All consecutive adult patients hospitalized in the participating hospitals (see Appendix) with SAB diagnosed between 1^st^ April 2009 and 31^st^ October 2011 were prospectively enrolled. SAB was defined as at least one blood culture bottle collected in a participating hospital and yielding *S*. *aureus*. Patients presenting with catheter colonization without SAB, defined as blood cultures positive only in bottles collected through a vascular access line were excluded.

Patients were examined and treated according to usual hospital procedures. Echocardiography was encouraged but was not mandatory for patient’s enrolment.

Trained research assistants prospectively collected clinical, biological and therapeutic data through a standardized case report form in each center. Clinical data included demographics, background characteristics (comorbidities, IE-predisposing conditions), healthcare contacts within the 90 days preceding hospitalization including invasive procedures, and setting of acquisition as defined below. SAB characteristics were recorded. These characteristics comprised presumed source of infection as defined by the clinician in charge of the patient and deep foci of infection (endocardium, lung and pleura, bones and joints, muscles, urine, central nervous system) as attested by imaging studies or positive culture of a normally sterile site. Detailed results of echocardiography were recorded, including regurgitation, vegetation, abscess and prosthesis dehiscence. Bacteriological data included susceptibility to main antistaphylococcal antibiotics and date and results of blood cultures. Therapeutic data included antibiotics, catheter removal, surgery, and admission to intensive care unit (ICU). All patients, their relatives or physicians were contacted 12 weeks after the beginning of the SAB to check if patients were alive, and survivors at week 12 were asked if they were able to engage in their usual activities using a five-item questionnaire.

### Setting of acquisition—definitions

Healthcare-associated SAB was defined as either nosocomial or non-nosocomial healthcare-associated infection [13]. Nosocomial SAB was defined as infection developing in a patient hospitalized for more than 48 hours prior to the onset of signs/symptoms consistent with bloodstream infection. Non-nosocomial healthcare-associated infection was defined as SAB diagnosed within 48 hours of admission in an outpatient with extensive healthcare contact as reflected by any of the following criteria: (1) received intravenous therapy, wound care, or specialized nursing care at home within the 30 days prior to the onset of SAB; (2) attended a hospital or hemodialysis clinic within the 30 days before the onset of SAB; (3) was hospitalized in an acute care hospital for 2 or more days in the 90 days before the onset of SAB; or (4) resided in a nursing home or long-term care facility. Community-acquired SAB was defined as SAB diagnosed within 48 hours of admission in a patient who did not fit the criteria for healthcare-associated infection.

### Infective endocarditis classification

A local adjudication committee made up of cardiologists, infectious diseases specialists and bacteriologists classified all SAB cases in each participating hospital as definite, possible or excluded IE according to the modified Duke criteria [22].

### Statistical analyses

Factors associated with definite IE according to modified Duke criteria were analyzed using multivariable logistic regression in the subpopulation of patients who were not referred to the participating hospital for the management of IE. Because our purpose was to identify patients with IE within the first 48 hours of care, we only included values that were present during the first 48 hours of SAB diagnosis. Similarly, we defined persistent bacteremia as positivity of blood cultures at 48 hours or later. Variables were selected to enter the model if they were associated with outcome with a p-value < 0.10 in bivariate analysis. A stepwise backward procedure was used to select the variables with a p-value < 0.05 in the final adjusted model.

We first performed the analysis in the whole population including predisposing heart conditions and setting of SAB acquisition as explicative variables. Secondly, as setting of acquisition and previously known IE predisposing conditions are known variables at the beginning of SAB care, we calculated the proportion of IE according to both setting of acquisition and predisposing conditions and then performed the analyses in sub-groups of patients: patients with or without previously known IE predisposing conditions, patients with community or non-nosocomial healthcare related, or nosocomial SAB.

Finally, in sensitivity analyses, we studied the factors associated with IE in the population of patients who underwent echocardiography, where only those patients fulfilling a major echocardiographic criterion in the modified Duke classification or having bacteriologically and/or histologically proven IE were classified as definite IE. Analyses were performed with SAS software (version 9.3).

The study is reported according to the STROBE statement for reporting observational studies [23].

## Results

### Study population

A total of 2091 patients with SAB were enrolled. After exclusion of the 83 patients referred to the participating hospital for the management of IE, 2008 patients were kept for the analyses. Main characteristics of patients are described in Table 1. Most patients had severe comorbidities including rapidly or ultimately fatal disease in 55.7%. Only 63 patients (3.1%) were past or current injecting drug users. Previously known IE predisposing heart conditions were reported in 24.4% of patients (prosthetic valve in 7% and native valve disease in 17.4%).

**Table 1:** Main characteristics of the 2008 patients with *Staphyloccus aureus* bloodstream infection enrolled in the VIRSTA study, 2009–2011.

Baseline characteristics	
Median age (years) (IQR)	70 (58–81)
Male gender	1295 (64.5%)
Diabetes mellitus	857 (42.7%)
Hemodialysis	211 (10.5%)
Immunodepression	738 (36.8%)
McCabe score:	
- ultimately fatal disease	751 (37.4%)
- rapidly fatal disease	368 (18.3%)
Injecting drug use	63 (3.1%)
Known heart disease predisposing to IE	
- none	1518 (75.6%)
- prosthetic valve	140 (7.0%)
- native valve disease	350 (17.4%)
Cardiac device*	217 (10.8%)
Presumed source of SAB	
- unidentified	406 (20.2%)
- skin	384 (19.1%)
- urinary tract	103 (5.1%)
- lung	122 (6.1%)
- peripheral venous line	143 (7.1%)
- central venous line	365 (18.2%)
- arterial line	19 (0,9%)
- arteriovenous fistula	42 (2.1%)
- intravenous drug use	35 (1.7%)
- surgery	303 (15.1%)
- other	86 (4.3%)
Presumed place of acquisition	
- unknown	58 (2.9%)
- community	522 (26.0%)
- non-nosocomial health care-related	353 (17.6%)
- nosocomial	1075 (53.5%)
Penicillin resistance	1808 (90.3%)
Methicillin resistance	381 (19.0%)
Complicated SAB	734 (36.6%)
Infective endocarditis	221 (11.0%)

Abbreviations: IQR: interquartile range; IE: infective endocarditis; SAB: *Staphylococcus aureus* bloodstream infection.

* Pacemaker or implantable cardiovertor defibrillator.

Presumed setting of acquisition was nosocomial in 1075 patients (53.5%), with an estimated incidence of nosocomial SAB of 0.159/1000 patients-days of hospitalization in the participating centers (95% confidence interval: 0.111–0.219). Frequency of methicillin-resistance was 19% in the whole population, 9.8% in community-acquired SAB and 22.3% in healthcare-associated SAB (21.7% in nosocomial, 24.4% in non-nosocomial).

### Deep foci of infection

At least one echocardiography was performed in 1348 patients (67.1%), among whom 605 (30.1% of the whole population) had at least one transesophageal echocardiography. Echocardiography was performed significantly more frequently in patients with IE-predisposing condition (data not shown). A deep focus of infection was detected in 36.6% of patients. Definite IE was the most frequent deep focus of infection, found in 221 out of 2008 patients (11.0%), followed by bone and articulations and lungs localizations. When restricting the population to the subjects who underwent echocardiography, frequency of IE was 15.6% (n = 210). Main characteristics of IE are described in Table 2 and main deep foci in patients with SAB without IE in Table 3.

**Table 2:** Description of the 221 infective endocarditis diagnosed among the 2008 patients enrolled in the VIRSTA study, 2009–2011.

Characteristics and outcome	n (%)
Median age (years) (IQR)	
Male gender	144 (65.2%)
Location of IE	
- aortic	39 (17.7%)
- mitral	61 (27.0%)
- aortic and mitral	21 (9.5%)
- right-sided	17 (7.7%)
- bilateral	20 (9.1%)
- cardiac device only*	7 (1.8%)
- undetermined	56 (25.3%)
Classification according to modified Duke criteria	
- surgically definite	24 (10.9%)
- clinically definite	197 (89.1%)
- 2 major criteria	139 (62.9%)
- 1 major criterion, ≥ 3 minor criteria	56 (25.3%)
- 5 minor criteria	2 (0.1%)
Severe sepsis without septic shock	56 (25.3%)
Septic shock	57 (25.8%)
Stroke	47 (21.2%)
Other embolic event	67 (30.3%)
*Staphylococcus aureus* meningitis	13 (5.9%)
Brain abscess	8 (3.6%)
Vertebral osteomyelitis	20 (9.0%)
In-hospital death	78 (35.3%)
12 week case-fatality rate	87 (39.4%)

Abbreviations: IQR: interquartile range; IE: infective endocarditis.

**Table 3:** Complication and outcome of the 1787 *Staphylococcus aureus* bloodstream infections without infective endocarditis, VIRSTA Study 2009–2011.

Characteristics and outcome	n (%)
Deep focus of infection	513 (28.7%)
- lung	141 (7.9%)
- urinary tract	94 (5.3%)
- vertebral osteomyelitis	48 (2.7%)
- prosthetic joint	66 (3.7%)
- other osteo-articular infections	110 (6.2%)
- myositis	38 (2.1%)
- central nervous system	16 (0.9%)
Severe sepsis without septic shock	208 (11.6%)
Septic shock	254 (14.2%)
In-hospital death	440 (24.6%)
12 week case-fatality rate	559 (31.3%)

### Follow-up

Main complications and outcomes are described in Table 2 for patients with IE and in Table 3 for patients without IE. Complications occurred more frequently in patients with IE. In-hospital global mortality was 25.8%, 24.6% in patients without IE vs 35.3% in patients with IE (p = 0.003). Mortality at 12 weeks was 32.2%, 31.3% in patients without IE vs 39.4% in patients with IE (p = 0.03). In survivors, inability to engage in usual activities 12 weeks after occurrence of SAB was common (56%), with no significant difference between patients with and without IE.

### Adjusted analyses of factors associated with IE

Table 4 presents the crude frequencies of IE according to main characteristics of patients, as well as crude and adjusted odds-ratios (aOR) in the models performed on the whole population of 2008 patients. Table 5 presents the crude proportion of IE according to both setting of acquisition and predisposing heart condition.

**Table 4:** Factors associated with Infective Endocarditis in the whole population, VIRSTA study 2009–2011.

Factor studied	All patients with SAB (n = 2008)	Patients with IE (n = 221) N (%)	Crude OR	95% CI	Crude p-value	Adjusted OR	95% CI	Adjusted p-value
**Baseline characteristics**								
Diabetes mellitus					0.002			
No	1441	196 (14)	1					
Yes	567	25 (4)	0.60	0.4–0.8				
Intravenous drug use					< 0.0001			< 0.0001
No	1945	197 (10)	1			1		
Yes	63	24 (38)	5.46	3.2–9.3		5.41	2.7–10.9	
Immunodepression					0.06			
No	1270	152 (12)	1					
Yes	738	69 (9)	0.76	0.6–1.0				
Known heart disease predisposing to IE					< 0.0001			< 0.0001
- none	1518	106 (7)	1			1		
- prosthetic valve	140	46 (33)	6.52	4.4–9.8		9.66	5.8–16.0	
- native valve disease	350	69 (20)	3.27	2.4–4.6		4.06	2.8–6.0	
Intracardiac device					0.0006			
No	1791	181 (10)	1					
Yes	217	40 (18)	2.01	1.4–2.9				
**Initial SAB characteristics**								
Presumed source					< 0.0001			
Unidentified	406	60 (15)	1.38	0.9–2.1				
Skin	384	43 (11)	1					
Intravenous drug use	35	18 (51)	8.40	4.0–17.5				
Urinary tract	103	13 (13)	1.15	0.6–2.2				
Lung	122	3 (3)	0.20	0.1–0.7				
Surgery	303	29 (10)	0.84	0.5–1.4				
Venous peripheral line	143	9 (6)	0.53	0.2–1.1				
Venous central line	365	24 (7)	0.56	0.3–0.9				
Arterial line	19	2 (10)	0.93	0.2–4.2				
Arteriovenous fistula	42	9 (21)	2.16	1.0–4.8				
Other	86	11 (13)	1.16	0.6–2.4				
Presumed place of acquisition					< 0.0001			< 0.0001
Community	522	107 (21)	3.76	2.7–5.2		2.88	1.9–4.3	
Non-nosocomial health care	353	42 (12)	1.97	1.3–3.0		2.22	1.4–3.5	
Hospital	1075	69 (6)	1			1		
Unknown	58	3 (5)	0.80	0.2–2.6		0.69	0.2–2.7	
Duration of symptoms before collection of blood culture					< 0.0001			
< 1 day	945	74 (8)	1					
> 1 day	983	142 (14)	1.99	1.5–2.7				
Unknown	80	5 (6)	0.78					
Penicillin resistance					0.35			
Yes	1808	195 (11)	1					
No	200	26 (13)	1.24	0.8–1.9				
Methicillin resistance					0.02			
Yes	381	30 (8)	1					
No	1623	191 (12)	1.56	1.0–2.3				
C-reactive protein > 190 mg/L					< 0.0001			0.0003
No	952	72 (8)	1			1		
Yes	929	141 (15)	2.19	1.6–3.1		1.91	1.3–2.7	
Missing value	127	8 (6)	0.78	0.3–1.7		0.76	0.3–1.7	
**Early events (available within the first 48 hours of diagnosis)**								
Severe sepsis or septic shock					< 0.0001			0.0005
No	1513	126 (8)	1			1		
Yes	495	95 (19)	2.61	2.0–3.5		1.89	1.3–2.7	
Stroke					< 0.0001			< 0.0001
No	1962	193 (10)	1			1		
Yes	46	28 (61)	14.3	7.7–26.2		7.50	3.3–16.8	
Other embolic event					< 0.0001			< 0.0001
No	1955	188 (10)	1			1		
Yes	53	33 (62)	15.5	8.7–27.6		10.5	5.0–21.8	
Meningitis					< 0.0001			< 0.0001
No	1986	208 (11)	1			1		
Yes	22	13 (59)	12.3	5.2–29.2		8.32	2.7–25.4	
Vertebral osteomyelitis					0.01			
No	1980	213 (11)	1					
Yes	28	8 (29)	3.32	1.4–7.6				
Persistent bacteremia at 48 hours					< 0.0001			< 0.0001
No	1664	136 (8)	1			1		
Yes	344	85 (25)	3.69	2.7–5.0		4.01	2.8–5.7	

Abbreviations: SAB: *Staphylococcus aureus* bacteremia; IE: infective endocarditis; OR: Odds-ratio; CI: confidence interval.

The following factors were not associated with IE in univariate analyses with a p > 0.10: age, sex, dialysis, McCabe score, penicillin sensitivity.

**Table 5:** Rate of IE according to predisposing heart disease and setting of acquisition.

Setting of acquisition	Predisposing heart disease			Total
	Yes, prosthetic	Yes, native	No	
**Community associated—intravenous drug use**	2/2 (100%)	1/3 (33.3%)	18/38 (47.4%)	21/43 (48.8%)
**Community associated—non intravenous drug use**	20/30 (66.7%)	31/80 (38.8%)	35/369 (9.5%)	86/479 (18.0%)
**Non-nosocomial healthcare associated**	6/13 (46.2%)	15/66 (22.7%)	21/274 (7.7%)	42/353 11.9%
**Nosocomial**	18/94 (19.1%)	20/191 (10.5%)	31/790 (3.9%)	69/1075 (6.4%)
**Unknown**	0/1 (0%)	2/10 (20%)	1/47 (2.1%)	3/58 (5.2%)
**Total**	46/140 (32.9%)	69/350 (19.7%)	106/1518 (7%)	221/2008 (11%)

The risk of IE was strongly associated with predisposing condition, i.e. heart disease and injecting drug use. However, the frequency of IE in patients without underlying heart condition was 7.0% globally and 3.9% among patients with nosocomial SAB. Globally, the proportion of patients with IE and without predisposing condition, was 39.8% (Table 5). We also observed that IE was more frequent in patients who acquired SAB outside the hospital as compared to nosocomial SAB, with aORs of 2.9 for community SAB and 2.2 for non-nosocomial healthcare-associated SAB in the whole-population analysis. These associations were observed in patients with and without underlying heart condition (data not shown).

Severe sepsis during the first 48 hours was independently associated with IE, as well as high levels of C-reactive protein. Duration of bacteremia > 48 hours was strongly associated with the presence of IE. Early embolic events and meningitis were also associated with IE.

The percentage of variability explained by the multivariable model was low, as attested to by the pseudo-R2 of 0.37 for the final model in the whole population. The sensitivity analyses performed in the subpopulation of the 1348 patients who underwent echocardiography and considering only definite IE defined surgically or echocardiographically, yielded similar results (data not shown).

## Discussion

In this large prospective study, SAB was associated with high morbidity and mortality. A deep focus of infection was identified in more than one third of patients, including IE in 11%, which was the most frequent complication and was associated with a poorer outcome. Frequency of IE varied according to predisposing heart condition, setting of acquisition with a decreasing gradient of frequency between community, outpatient healthcare and hospital, and to some parameters available at the early stage of management including C-reactive protein and duration of bacteremia. A high proportion of IE occurred in patients who had no known predisposing condition.

Our study is the largest prospective cohort of SAB reported to date [9,24–25]. We comprehensively enrolled consecutive patients, which reduced the risk of selection bias. The study was performed in eight different hospitals located in different regions in France; results should thus be more generalizable than those yielded in a monocentric study. Thus, we believe that this study provides accurate estimates of the risk of IE in case of SAB in various settings and among patients with various predisposing conditions in high-income countries.

The incidence of nosocomial SAB was high in the 8 participating centers, albeit similar to that observed in other high-income countries [26,27]. Healthcare-related SAB were the most frequent, representing 70% of cases in our study, a proportion similar to that reported in other contemporary studies [3,4,9]. Thus, it seems essential to improve the prevention of this dreadful complication of medical progress. In Australia, implementation of non-specific infection prevention and control initiatives was associated with a substantial reduction of the incidence of nosocomial SAB [28]. Recent studies have suggested that universal decontamination may also be useful, especially in the ICU [29,30]. Approximately one in five SAB in our study were healthcare-associated but non-nosocomial. The use of outpatient invasive medical therapy is increasing and infection control is difficult to implement in this setting. The impact of preventive measures, such as *S*. *aureus* nasal decontamination in patients receiving outpatient intravenous therapy should be a matter of clinical research.

We observed IE of 11% of our population. As echocardiography was performed in only 60% of study patients, this estimation is probably conservative. In studies with more systematic use of echocardiography, the proportion of IE was higher, around 20% [9,20,31]. However, underestimation of IE was probably not major in our study, as the proportion of IE was not much higher (i.e. 15.6%) in the subgroup of patients who underwent echocardiography. The difference in rates of IE reported in the literature may be due to differences in proportion of at-risk populations enrolled in the studies. In fact, the frequency of IE varies widely across strata of risk factors. For example, in our study, IE frequency varied from 38% in injecting drug users and 33% in patients with prosthetic heart valve, to 4% in patients with nosocomial SAB in the absence of underlying heart condition, the strata of patients with the lowest rate of IE.

We observed a quite low rate of deep foci of infection other than IE in this cohort, compared to other cohort studies of SAB [10]. This may be the consequence of the high proportion of nosocomial SAB in our study. Nosocomial SAB originate mostly from venous access and are usually diagnosed and managed early, which may lead to a lower risk of metastatic spread of infection compared to SAB occurring outside the hospital who may be referred later in presentation. This is also a plausible explanation for the lower risk of IE in patients with nosocomial SAB.

Among factors associated with IE we observed, most are longtime known, they are predisposing conditions to IE like heart valve disease or injecting drug use or direct complication of IE like stroke. Some factors have been identified more recently in smaller studies [17,25,32,33]. Among the latter, the most striking is persistent bacteremia, which was associated with a higher frequency of IE whatever the setting of acquisition and the presence of IE predisposing condition. Such an association between duration of bacteremia and IE may be either a cause or a consequence of IE. Nevertheless, this finding emphasizes the necessity of systematically draw follow-up blood cultures during the first days of management of SAB.

A finding which has not been previously reported to our knowledge is the higher frequency of IE in non nosocomial healthcare-related SAB compared to those acquired in the hospital. The decreasing gradient of frequency of IE complicating SAB between community, outpatient healthcare and hospital may be explained by (i) a higher proportion of identifiable and removable primary focus, mostly venous access, in outpatient healthcare-related infections compared to community-acquired ones and (ii) a later diagnosis of infection in outpatients compared to inpatients. Earlier management of bacteremia may also be an explanation for the lower rate of IE observed in unadjusted analyses in patients with diabetes mellitus or immunodepression. We also found higher C-reactive protein in the first 48 hours as associated with IE. This biological marker may thus be useful in the early management of SAB, indicating when elevated a higher risk for complicated infection, possibly through a higher bacterial load. Lastly, meningitis is rare during SAB but very frequently associated with IE and appears thus to be a useful clue in clinical care.

Nevertheless, our multivariable model did explain only a low proportion of the variability, which suggests that many factors favoring a location in the endocardium in case of SAB still remain to be discovered. This point is also illustrated by the fact that 40% of IE occurred in patients without predisposing condition and by the relatively high proportion of IE in patients without underlying heart disease (7%), even in case of nosocomial acquisition of bacteremia (4%). The impact of bacterial genotype or virulence factors has still to be better understood [34–36]. Impact of host genetic background has never been studied to our knowledge. Moreover, because most factors associated with IE are not strongly discriminant, it seems rather difficult to rule out IE in a patient with SAB without actively searching for IE by performing transthoracic or transesophageal echocardiography. An ecological study performed in the Netherlands recently suggested that routine use of echocardiography in case of Gram-positive bacteremia is associated with a higher rate of IE diagnosis and a lower risk of death in case of IE, which suggests that early detection of IE is beneficial [20]. Indeed, the 2009 European guidelines on IE diagnosis, prevention and treatment recommend performing echocardiography in all patients with SAB [37]. By providing estimates of frequency of IE in case of SAB, our data may help the clinician on the decision to perform this exploration.

This study has some limitations. First, patients were enrolled in tertiary care centers. This has probably led to the recruitment of more severe patients with a higher prevalence of comorbidities leading to a possible overestimation of frequency of poor outcomes. However, we minimized the referral bias by excluding from analyses of risk factors for IE those patients referred for management of IE. Second, as the study was observational, we could not control for any factor.

Our findings underscore the need to promptly detect and manage deep foci of infection when SAB is diagnosed. Among those foci, IE is the most frequent. The frequency of IE varies widely according to setting of acquisition and medical conditions but is not associated with any known predisposing condition in 40% of patients. It seems thus important to look for the association between *S*. *aureus* IE and yet unknown factors like host and bacterium genetic background. The prevention of SAB is an urgent goal for infection control in hospital and outpatient healthcare.
